# Improving access to quality medicines in East Africa: An independent perspective on the East African Community Medicines Regulatory Harmonization initiative

**DOI:** 10.1371/journal.pmed.1003092

**Published:** 2020-08-12

**Authors:** Alexander R. Giaquinto, Alberto Grignolo, Lawrence Liberti, John C. W. Lim, Tomas Salmonson, Fernand Sauer, Henrietta Ukwu

**Affiliations:** 1 ARG Consulting, LLC, United States of America; 2 Parexel, Billerica, Massachusetts, United States of America; 3 Centre for Innovation in Regulatory Science, London, United Kingdom; 4 Centre of Regulatory Excellence, Duke—NUS Medical School, Singapore; 5 Consilium Salmonson & Hemmings, Uppsala, Sweden; 6 Académie Nationale de Pharmacie, Paris, France; 7 Otsuka Pharmaceutical Companies, Princeton, New Jersey, United States of America

## Abstract

Alexander Giaquinto and co-authors discuss the East African Community's Medicines Regulatory Harmonization initiative.

**Summary points**

- The goal of improving access to quality medicines in East Africa has been addressed by the East African Community (EAC) Medicines Regulatory Harmonization (MRH) initiative, which is working to simplify the process of registering medicines and increase the speed at which registration applications are reviewed while ensuring that only high-quality medicines are approved.
- This article was written by a group of authors with varied expertise in global regulatory affairs but no involvement to date in the EAC MRH initiative. The authors were asked to provide an independent perspective on the initiative’s work since its launch in 2012, as well as its plans for the future.
- The authors believe that the strengths of the EAC MRH’s initiative include its rapid implementation of a joint product assessment process, by which a manufacturer can submit a single marketing authorization application and receive a decision respected by all EAC member states, as well as the creation and use of a common technical document (CTD) for registration applications that is accepted by all member states. In addition, the initiative’s twinning program, in which more mature national regulatory authorities carry out joint activities alongside less mature authorities, is an excellent way to build the capacity of all partner states.
- The authors believe the EAC MRH initiative may be able to further improve access to essential medicines by increasing transparency, inviting and responding to feedback from industry partners, consistently meeting its advertised assessment and registration timelines, streamlining the joint product assessment process, taking advantage of work done by peer institutions, focusing on the activities most likely to have the greatest public health return, and using metrics and benchmarks to identify opportunities for greater efficiency.
- The initiative may be able to maintain and even expand its activities by charging industry user fees. This will become increasingly important as the scope of the initiative grows to include a greater emphasis on pharmacovigilance activities and, as more medicines undergo the joint approval process, handling the postapproval regulatory burden.
- Strengthening the EAC MRH’s legal framework, such that member states are legally required to respect joint regulatory decisions instead of being bound only by goodwill, will also be important for securing the initiative’s future.

In the final article in this Special Collection in *PLOS Medicine* about the East African Community (EAC) Medicines Regulatory Harmonization (MRH) initiative [1–4], we, a group of authors who have had no involvement in the EAC MRH’s work to date, provide an independent perspective on the program’s work thus far and its plans for the future. The initiative’s goal is to improve access to essential medicines in East Africa by simplifying the process of registering medicines and increasing the speed at which registration applications are reviewed while ensuring that only high-quality medicines are approved. Here, we share our thoughts on the global context in which this initiative operates, highlighting successful practices from around the world that may be helpful to the EAC MRH initiative as it moves forward. In turn, we call attention to some of the EAC MRH initiative’s practices and achievements that may be helpful to other harmonization initiatives.

We will begin by explaining how this article was written. Representatives of the Bill & Melinda Gates Foundation, one of the EAC MRH initiative’s partners, asked one of us (A. Grignolo) to organize and deliver this independent perspective on the initiative. Dr Grignolo then invited us to contribute, as a team of coauthors with rich and varied experience in regulatory harmonization. We were provided with drafts of the other articles in the Special Collection [1–4], but otherwise, this perspective was written without input or influence from the other authors or from the EAC MRH initiative or its partners, including the Bill & Melinda Gates Foundation. Information about each author’s background can be found in S1 Text.

## The function of regulatory agencies

Regulatory agencies and programs such as the EAC MRH initiative must carry out 2 fundamental tasks: protecting the public against harm and promoting public health. The need for regulatory agencies to perform these functions was dramatically highlighted in the 1960s, when the United States Food and Drug Administration (US FDA) blocked thalidomide’s approval in the US, preventing the severe birth defects that had occurred in many other countries [5]. This incident inspired the Kefauver-Harris Drug Amendments Act in the US and similar legislation in other countries requiring drug manufacturers to prove that medicines are safe and effective before they can be marketed. Today, regulatory agencies across the world also require that drug manufacturers and clinicians report serious adverse events according to strict timelines before and after a medicine’s approval. In addition to analyzing these adverse event reports, regulatory agencies perform a variety of other pharmacovigilance activities to ensure that the medicines available on the market are safe and of high quality [6,7]. Here, we describe some of the best practices that regulatory agencies have identified for carrying out their key functions in hopes that some of these practices may be useful to the EAC MRH initiative as it takes its place on the world stage.

## Best practices from the European Union’s regulatory system

We begin with the European Union (EU)’s regulatory system, arguably the world’s largest MRH program and the one most relevant to the EAC MRH initiative. The EU system oversees the regulation of medicines across some 30 European countries. It has existed in its current state since 1995 and provides 4 pathways by which drug manufacturers can apply for marketing authorization (Table 1) [8]. The EU system consists of (1) the European Commission, which implements and oversees the legal basis for the system, ensuring that member states recognize the system’s decisions; (2) the European Medicines Agency (EMA), which oversees and coordinates the assessment of new medicines through the centralized procedure; and (3) the national agencies of EU member states, which carry out scientific assessments regardless of which marketing authorization procedure is used. The system’s decentralized procedure—in which an EU member state performs an initial evaluation of a medicine, which is then vetted by the other member states—is most akin to the EAC MRH’s joint assessment program.

**Table 1:** Four pathways to obtain marketing authorization for medicines in the EU.

Pathway	Process	Notes on use
Centralized procedure	Manufacturer submits application directly to the EMA. The CHMP then draws on scientific expertise from EU member states (which collectively contribute to the scientific evaluation of new products) to determine whether the medicine should be authorized.	Mandatory for many new substances
Decentralized procedure	One EU member state performs the initial evaluation of a medicine and issues a draft assessment report, after which other member states have the opportunity to ask questions and raise objections.	Most generics in the EU are approved via these procedures
Mutual recognition procedure	Similar to the decentralized process; if a medicine has already been approved in at least 1 EU member state, and its manufacturer is seeking approval in at least 1 other member state, the member state that has already authorized the medicine issues a draft assessment report. Afterwards, other member states have the opportunity to ask questions and raise objections.	
National procedure	A medicine is approved in only a single EU member state and no interaction with or recognition by other member states occurs.	Rarely used

**Abbreviations:** CHMP, Committee for Medicinal Products for Human Use; EMA, European Medicines Agency; EU, European Union

Several best practices emerged early in the history of the EU’s system and have stood the test of time. First, transparency is crucial in gaining the trust and approval of stakeholders both within and outside of the harmonization network. Creating groups of experts that work together transparently not only builds trust between assessors from different countries but also ensures that similar scientific standards are being applied systemwide. For example, the Working Parties of the EU’s Committee for Medicinal Products for Human Use (CHMP) bring together experts in particular scientific fields, such as quality, biologics/biosimilars, or oncology [9]. The Working Parties draft scientific guidance documents, help evaluate marketing authorization applications submitted via the centralized procedure, and share their guidance and decisions—including the underlying reasoning—with the public. Indeed, it is essential for a harmonization program to describe all regulatory processes in detail in a form readily accessible to all stakeholders. The EMA provides a great deal of information on its website, including scientific guidelines and assessment reports, which contain comprehensive descriptions of its product evaluations [10]. In addition, public debates in which multiple viewpoints can be discussed and public forums for gathering comments are extremely valuable; decisions made in closed-door meetings cannot secure the broad buy-in needed to make harmonization programs successful.

Currently, it is difficult to find sufficient detailed information about the EAC MRH initiative’s regulatory processes, including on the program’s website; in the absence of such information, industry users may be less likely to use the initiative’s new joint regulatory processes, as reported in a recent survey about the program [11]. In addition, it is unclear who has the opportunity to comment on the regulatory guidelines developed by the initiative’s working groups. Therefore, there may be areas in which the initiative can increase transparency.

A second best practice is the involvement of the pharmaceutical industry. To be clear, the EU’s system exists to regulate industry. However, the system also needs industry to produce medicines and comply with regulations, and industry representatives have often provided valuable feedback about how to improve regulatory processes. Industry can thus be viewed as a “partner” in the effort to make regulatory assessments more efficient, predictable, and credible. Beyond the EU, different regional harmonization programs have identified different roles for industry. For example, the Association of Southeast Asian Nations (ASEAN)’s Pharmaceutical Products Working Group invites industry representatives to attend dedicated sessions held prior to when regulators have their meetings and also allows industry representatives to attend regulators’ meetings as observers. In the Asia-Pacific Economic Cooperation (APEC)’s Life Sciences Innovation Forum–Regulatory Harmonization Steering Committee, industry plays a more active role, joining in discussions and helping to prioritize work areas. Finally, the time, funding, resources, and motivation of industry members played a critical role in the early successes of the International Council for Harmonisation of Technical Requirements for Pharmaceuticals for Human Use (ICH; originally the International Conference on Harmonisation of Technical Requirements for Registration of Pharmaceuticals for Human Use), including establishing the Common Technical Document (CTD) and numerous guidelines on product quality, safety, and efficacy. Industry members, who had (and still do have) seats on the ICH Steering Committee, drew on their deep knowledge of regulatory processes to help select which regulatory guidelines to work on and to help create and refine those guidelines. Although ICH is not a regional harmonization program, this example highlights the potential of industry representatives to identify important regulatory inefficiencies and also potential solutions.

Within the structure of the EAC MRH initiative, industry representatives do not seem to play a visible role. It is also unclear whether a system is in place for routinely obtaining feedback from industry users regarding the program’s joint assessment or inspection processes. The EAC MRH initiative may want to consider how industry entities, such as the Federation of East African Pharmaceutical Manufacturers, could play a more meaningful role going forward, without foregoing their accountability as regulated entities. Legislation and enforcement can play an important role in preventing undue influence by industry. For example, the US Foreign Corrupt Practices Act (FCPA) forbids US companies, including drug manufacturers, from offering or paying bribes to foreign officials in any part of the world to further their business [12]; violating this law results in severe consequences. In the EAC, local laws and regulations could be leveraged to impose appropriate penalties against drug manufacturers who seek to take unfair advantage of regulators and the regulatory process through illegal actions. For instance, if the EAC MRH initiative eventually passes legislation establishing government-sanctioned user fees to support the timely and thorough evaluation of marketing applications, it could use that very same legislation to outlaw and punish bribery, as has been done with similar laws elsewhere in the world.

A third, and related, best practice is making joint regulatory processes attractive to industry users, who value reliability, efficiency, and convenience. The EU’s system gained and retains industry’s support in large part because of its ability to complete high-quality assessments of marketing authorization applications in less than 1 year, paving the way for licenses that are valid across Europe. This is a much more attractive prospect than the lengthy wait time that companies previously experienced [13]. Since the EAC MRH initiative’s joint assessment program launched in 2015, it has received 93 product applications (and assessed 83 of them)—a solid start but one suggesting that the process has yet to be fully embraced by industry [3]. The program’s attractiveness to users may be limited by its apparent inability to meet target timelines. For example, many EAC partner states are not registering jointly approved products within the advertised 3-month period [3]. An agreement by which all partner states are required to abide by joint assessment decisions would minimize this problem. Currently, however, EAC partner states have no such legal obligation and are, instead, motivated only by goodwill. Once the initiative demonstrates that it reliably meets its deadlines and its decisions are respected throughout the region—preferably by making data from external audits public—industry users will be likely to participate in greater numbers.

A final best practice is minimizing bureaucracy and streamlining decision-making, especially in the early stages of a harmonization initiative. When the EMA was founded, its Secretariat consisted of only 16 people. The current structure of the EAC MRH initiative, which appears to contain multiple levels of hierarchy and a large number of individuals [2], may hamper its ability to make decisions quickly and respond to problems in an agile manner. Perhaps, in establishing the planned EAC Medicines Agency, the EAC can reduce bureaucratic barriers to action. We believe it is a good idea to create this regional agency as soon as possible and to populate it with the top experts from national agencies; the longer this takes, the more reluctant the EAC’s national authorities may be to relinquish power to a regional authority.

## Best practices from other initiatives around the world

The EU’s system is only one of many regulatory harmonization initiatives from around the world [14]. A number of new initiatives have emerged recently inside Africa [1], and many prominent initiatives exist outside Africa as well (Table 2). In our work with these and other initiatives, a few best practices have emerged that are consistent with the maxim “simplify, streamline, and standardize.” These practices help programs improve efficiency and avoid devoting time and effort to duplicative processes that do not appreciably improve patient health.

**Table 2:** Some regulatory harmonization initiatives from around the world.

Inside Africa	Outside Africa	Global
• Arab Maghreb Union • EAC’s MRH Initiative • Economic Community of Central African States—Organization of Coordination for the Fight Against Endemic Diseases in Central Africa • Economic Community of West African States—Union Economique et Monétaire Ouest Africaine • Intergovernmental Authority on Development—Community of Sahel-Saharan States • South African Development Community—Common Market for Eastern and Southern Africa, including the ZAZIBONA Collaborative Medicines Registration initiative	• APEC’s Life Sciences Innovation Forum–Regulatory Harmonisation Steering Committee • ASEAN’s Pharmaceutical Product Working Group and Medical Device Committee • EU System, including the EMA • Gulf Health Council • Pan-American Network for Drug Regulatory Harmonization	• ICH

**Abbreviations:** APEC, Asia-Pacific Economic Cooperation; ASEAN, Association of Southeast Asian Nations; EAC, East African Community; EMA, European Medicines Agency; EU, European Union; ICH, International Council for Harmonisation of Technical Requirements for Pharmaceuticals for Human Use; MRH, Medicines Regulatory Harmonization

One best practice is to fully leverage available external resources and to learn from each other. The ICH website currently features 57 quality, safety, efficacy, and multidisciplinary technical guidelines that have been jointly developed with industry participation and adopted by regulatory authorities worldwide [15]. These guidelines aim to reduce duplicative testing and streamline the global development and registration of new medicines. In addition, the EMA has made available nearly 1,400 public assessment reports for human medicines [16], and the European Pharmacopeia [17] and US Pharmacopeia [18] websites contain extensive information on standards for high-quality medicines. These actions contribute to shared learning, transparency, and joint progress.

In recent years, the EAC MRH initiative has spent much of its time developing an EAC-specific CTD format for marketing applications in lieu of using ICH’s CTD format. It has also focused on creating EAC-specific regulatory guidelines and standard operating procedures. Although the EAC may need to modify existing global regulatory standards somewhat to ensure that its unique needs are met, introducing changes also increases the risk that harmonization with regulatory practices elsewhere in the world will be compromised. This could potentially delay access to needed medicines for EAC patients. In addition, time spent on such modifications cannot be spent on other activities that may have a greater public health return (for example, ensuring the quality of medicines, especially generic drugs, manufactured in or imported into the EAC).

Given the vast workload faced by the EAC MRH initiative, as well as by other regulatory programs across the world, collaborating with peer institutions has become a necessity. Could the initiative deliver a greater public health benefit if it started relying more on existing product assessments carried out by trusted regulatory authorities, as urged by the World Health Organization (WHO) [19] and practiced by other regulatory authorities [20,21] (Box 1)? This could free up the EAC MRH initiative’s time and resources to focus on which aspects of the assessments done by other agencies are relevant to EAC countries, as well as on carrying out more good manufacturing practice inspections and pharmacovigilance activities. The EAC MRH initiative has already decided to focus its joint assessments on products that are not eligible for WHO’s Prequalification Programme; this is a great example of using reliance to liberate resources for other important activities.

**Box 1. The concept of reliance in the regulation of medicines**

**Definition of regulatory reliance**

Reliance is defined by WHO as “an act whereby a regulatory authority in one jurisdiction may take into account or give significant weight to work performed by another regulator, or other trusted institution, in reaching its own decision” [19].

**How regulatory reliance works**

In practice, reliance can take many forms, such as establishing a recognition protocol by which the decisions of trusted regulatory authorities (such as WHO’s Prequalification Programme, the US FDA, or the EU’s medicines assessment and licensing system) are routinely accepted or developing an abridged review process for products already approved by those institutions [20,21].

**Best practices for regulatory reliance**

Currently, WHO is developing Good Registration Practices guidelines, including a component on Good Reliance Practices to aid regulators who are seeking to implement reliance models.

Another best practice is to focus the scope of a program’s activities on those most likely to have the greatest public health return. One important example would be the EAC MRH initiative’s plans to begin assessing biologics and biosimilars in the near future. Evaluating applications for these complex, expensive medicines requires a great deal of time and expertise. On the one hand, the more complex an application is, the more suitable it is for a regulatory pathway that pools expertise, such as the EU’s centralized procedure (mandated for such applications) or the EAC’s joint assessment program. On the other hand, given the resources required to assess these applications, perhaps it would be helpful if, when possible, the EAC relied on a trusted regulatory authority’s previous work when making decisions about biologics and biosimilars. This could allow the initiative to focus on registering medicines with a greater potential public health impact, such as affordable generic drugs, small-molecule new molecular entities, or even local versions of biosimilars. By contrast, the initiative’s plan to broaden the scope of its activities to regulating regional clinical trials has the potential to provide substantial benefits to EAC residents. If conducted properly and ethically, clinical trials could improve access to medicines for EAC residents, both in the short term for trial participants and in the long term for the broader population, which will benefit from the knowledge gained.

A final best practice is to use metrics and benchmarks to identify roadblocks and perform comparisons with peer institutions, so programs can become more efficient [21]. Collecting metrics can be challenging, especially for resource-strapped programs. It may be difficult to justify collecting data (on the time spent assessing an application, for example) whose public health impact is not immediately obvious. A program may also find the idea of collecting and publishing metrics of its own performance daunting when it is new or struggling, for fear the resulting data could prove “embarrassing.” However, early metrics should be viewed as a baseline; when they start to improve, so does the morale of program staff and the trust that outside partners have in a program.

**Fig 1:**
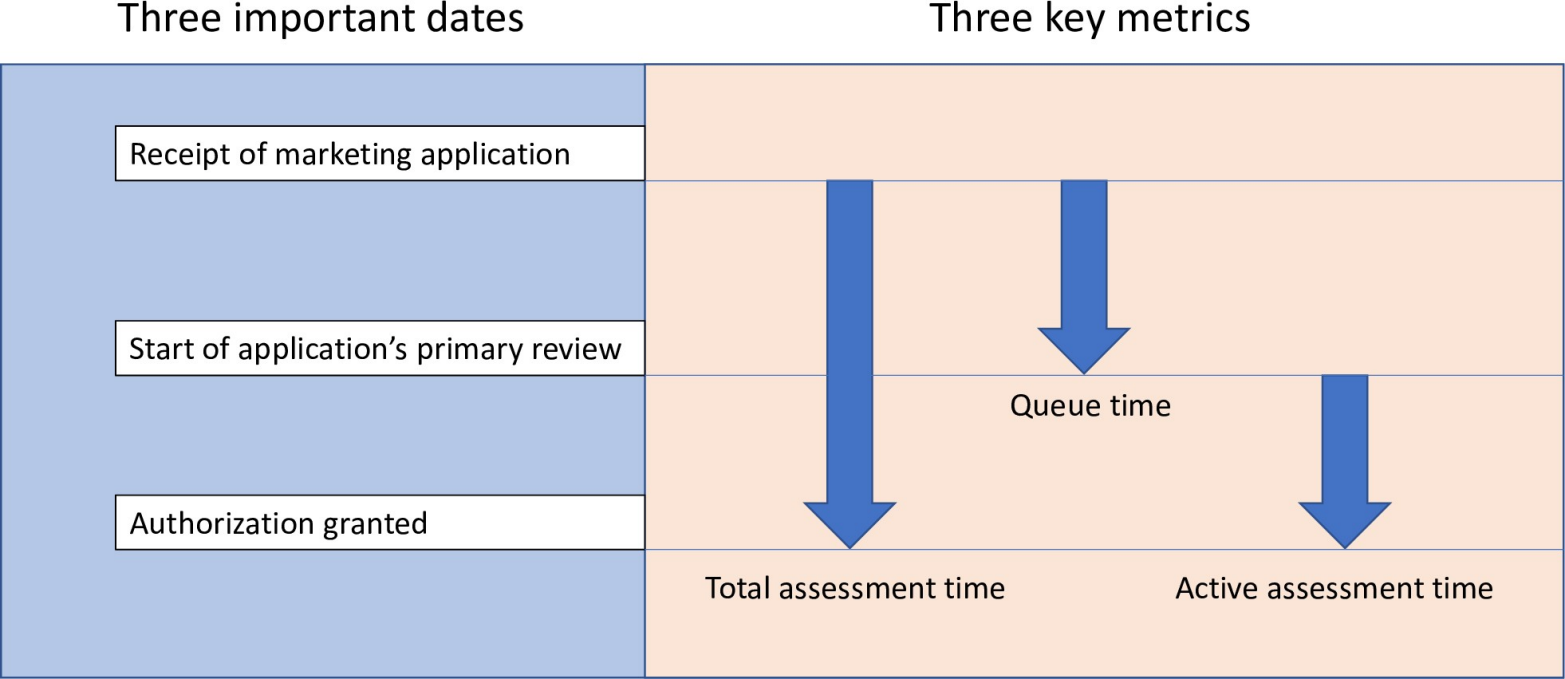
Metrics can help regulatory authorities improve their processes for assessing medicines. In particular, collecting data on 3 important dates in the medicine assessment process allows authorities to gain essential insight into problem points and improve their processes.

We believe all regulatory authorities should collect a few key metrics (Fig 1), and we provide several real-world examples of these metrics at work. In 2000, Health Canada was experiencing lengthy delays in product approvals. Analysis of the key metrics in Fig 1 revealed that a long queue time (approximately 200 days) resulted in a large backlog of applications. By implementing changes targeting queue time, Health Canada reduced its backlog by 99% between 2000 and 2006 and halved overall application approval times [22]. In the early 2000s, Japan’s Pharmaceutical and Medical Devices Agency (PMDA) was approving drugs roughly 2.5 years after the US and EU. After analyzing key metrics, the Japanese government revised the country’s Pharmaceutical Affairs law, dedicated more staff to assessments, and invested in regulatory skill building [22]. PMDA approval times are now on par with those of the US FDA [22]. Finally, the US FDA has kept detailed metrics on submissions and approvals for decades, as the Prescription Drug User Fee Act of 1992 and its subsequent renewals require the agency to set and achieve annual goals based on the previous year’s metrics. All of these organizations were able to speed up the assessment process without abandoning their primary focus on quality, safety, and efficacy by using metrics to identify and address the points in the system where unnecessary delays were occurring.

The EAC MRH initiative is already tracking time from application receipt to approval, or total assessment time [3]. By continuing to define target timelines for key milestones in the joint assessment process, developing formal processes for routinely monitoring relevant metrics, and making the resulting data public [21], the EAC MRH initiative can increase its efficiency and promote trust among stakeholders. Establishing a framework in which timely, high-quality assessments are the norm will result in safe and effective medicines reaching the market sooner, while unsafe or ineffective medicines are promptly denied approval.

## Best practices from the EAC MRH initiative

Several best practices have already emerged from the EAC MRH initiative. First, the commitment to regulatory harmonization demonstrated by the heads of the EAC’s national medicines regulatory authorities is impressive. Many regional medicines regulatory harmonization initiatives start as offshoots of larger pushes for trade and economic harmonization; the impetus for the EAC MRH initiative clearly came primarily from a concern with improving health. This commitment is reflected by the support for regional activities provided by the initiative’s dedicated Regional Technical Officers. Other harmonization initiatives have been stymied by a lack of such personnel. For example, whereas the EAC MRH initiative has jointly assessed 83 product applications since its launch in 2012 [3], ASEAN—which has been active for 20 years and began a pilot joint assessment program in coordination with WHO in 2017—has conducted only 1 joint assessment so far, although there is commitment to strengthening technical support for this initiative. The EAC regulatory authorities’ commitment to using the standardized CTD format has also helped reduce national assessment times from roughly 24 months at baseline to 8 to 14 months in Kenya and 10 to 12 months in Tanzania [3]. The amount of progress that the EAC MRH initiative has made in a few short years highlights the importance of adequate funding and dedicated personnel.

The EAC MRH initiative has other well-thought-out features. For example, the EAC’s twinning program, in which more mature national regulatory authorities carry out joint activities alongside less mature authorities, is an excellent way to build the capacity of all partner states. We are also impressed by the initiative’s decision to assign 2 teams to each joint assessment or evaluation. “Healthy cooperation and friendly competition” between regulatory authorities ensures high-quality, consistent assessments and also supports the continued education of assessors. The program also appears to enjoy a high degree of involvement from all partner states. Small countries have the potential to make outsized contributions to regional harmonization initiatives (for example, a senior drug-quality expert from Luxembourg has been a tireless and highly respected contributor to the EU’s regulatory system for many years). By actively involving both large and small partner states, the EAC MRH initiative is poised to capitalize on regional talent, wherever it comes from. Finally, the initiative has an unusually clear roadmap for the future, which will make it easier to stay focused on the program’s most important goals.

Some unique aspects of the context in which the EAC MRH initiative operates may make it difficult for other programs to replicate its results. A common problem across regional harmonization initiatives is that partner states have extremely different capacity levels. For example, APEC’s members include economies as diverse as those of Mexico, China, the US, Brunei, and Papua New Guinea. In addition, like the EAC MRH initiative, many regional harmonization programs lack a binding legal framework (such as exists in the EU) that requires partner states to abide by joint decisions. Therefore, joint assessment and inspection programs hinge on the level of trust between member states, and more mature regulatory authorities may be reluctant to recognize decisions that rely on the work of less mature authorities. The EAC MRH initiative has encountered this problem to some extent and has put a great deal of effort into building the capacity of each of its partner states. However, the EAC’s partner states have also been willing to grant one another an extraordinary amount of trust, perhaps due to their tight linguistic, historic, and cultural ties.

## Next steps for the EAC MRH initiative

The EAC MRH initiative can boast of some remarkable accomplishments since its launch in 2012. Here, we share our thoughts about some of its ambitious plans for the future.

In terms of funding, we believe that if the initiative can demonstrate that it meets its target timelines and its decisions are respected throughout the EAC, the pharmaceutical industry will be willing to pay substantial, legally authorized user fees, which can provide a stable source of income to sustain the program’s ongoing operations. This approach has been extraordinarily successful in the US for nearly 30 years (via the Prescription Drug User Fee Act); the EMA and PMDA also receive financial support from industry user fees. In addition to supporting the desired EAC Medicines Agency, user fees could be employed to compensate the national regulatory authorities that participate most in joint activities. For example, the EMA pays a portion of the industry user fees for each application to the national regulatory authorities that provide their expertise during assessment.

We also applaud the initiative’s plans to place a greater emphasis on pharmacovigilance, given the scope of the problem of substandard and counterfeit drugs in the EAC. As mentioned above, we believe it might make sense for the initiative to allocate even more of its resources to such work and to monitor the physical quality and integrity of the drug supply, though we recognize that much of the policing of drug safety must be done by individual countries. EAC countries should also consider joining MEDICRIME, the first international treaty against falsified medical products and devices involving threats to public health. It has been ratified by 12 European countries, as well as Guinea, Burkina Faso, and Ivory Coast [23].

In moving forward, the EAC MRH initiative may also want to consider how it will handle the postapproval regulatory burden (i.e., managing consistency across labeling changes) for jointly approved products, as the work required to regulate variations for new drugs is considerable. In addition, implementing reliance models would help the initiative maximize its public health returns, though the program will need industry’s cooperation to identify information that has been updated since previous application reviews by trusted regulatory authorities, as well as EAC-specific aspects of applications.

As the EAC MRH initiative takes its place alongside other regulatory harmonization initiatives, we welcome it and encourage it to stay the course. History has shown that it takes decades of patience and perseverance to set up a mature system. Though the EMA was established in 1995, the legal framework for pharmaceutical regulation in the EU had been established in 1965 (Council Directive 65/65). Similarly, after the concept of the CTD was first proposed at ICH, it took 2 years and a costly survey before the topic was accepted for development and an additional 3 years before the CTD guideline was issued. To ensure that the EAC MRH initiative has the staying power needed to improve access to medicines for the people of East Africa, we believe an essential next step is establishing a binding legal framework that lays out clear decision-making processes and obligates partner states to abide by program decisions. The initiative has strong support from national and regional leaders now, but this support may wax and wane over time in the absence of a binding legal framework. It is unlikely that the EU’s system could have survived and succeeded for decades, even as controversies arose among member states, without robust legal backing.

Given adequate legal, operational, and financial support, the EAC MRH initiative can help pioneer novel regulatory roles for Africa on the global stage. For example, medicines for health conditions that are primarily found in Africa are best evaluated in African countries, where the experts with the most experience with those conditions reside. In the future, the entire world may rely on African regulatory authorities to assess these types of medicines. Moreover, if the trials testing these medicines are conducted in Africa and are supported by appropriate local regulations, the patients who participate have the potential to benefit tremendously—not only from the development of effective new medicines that address important health problems in Africa, but also from the access to the health system that is provided by enrolling in a clinical trial.

Finally, the EAC MRH initiative’s work will help determine whether the African Medicines Agency becomes a reality as well as the shape this new regulatory authority will take [24,25]. With Africa’s steadily increasing population, developing efficient and competent regulatory mechanisms is becoming more important than ever, and the African Medicines Agency may have an important role in this work. This is truly an exciting time for MRH in Africa.

## Supporting information

S1 Text Author biographies. (DOCX)

## Abbreviations

APEC: Asia-Pacific Economic Cooperation
ASEAN: Association of Southeast Asian Nations
CHMP: Committee for Medicinal Products for Human Use
CTD: Common Technical Document
EAC: East African Community
EMA: European Medicines Agency
EU: European Union
FCPA: Foreign Corrupt Practices Act
ICH: International Council for Harmonisation of Technical Requirements for Pharmaceuticals for Human Use
MRH: Medicines Regulatory Harmonization
PMDA: Japan’s Pharmaceutical and Medical Devices Agency
US FDA: United States Food and Drug Administration
WHO: World Health Organization
