# Supplementary material for: Improving access to quality medicines in East Africa: An independent perspective on the East African Community Medicines Regulatory Harmonization initiative
Source: PLoS Med. 2020 Aug 12;17(8):e1003092. doi: 10.1371/journal.pmed.1003092 (PMC7423065; doi:10.1371/journal.pmed.1003092)
Supplement: S1 Text — (DOCX) [file pmed.1003092.s001.docx]

**Supporting Information**

**Improving access to quality medicines in East Africa: An independent perspective on the East African Community’s Medicines Regulatory Harmonization initiative**

**Author Biographies**

**Alexander R. Giaquinto, PhD**

Dr Giaquinto began his career in the pharmaceutical industry as a senior scientist with Vicks Division, Research and Development, Mount Vernon, New York. Over the years, he held many positions at Schering-Plough Corporation, including senior vice president of Schering-Plough Research Institute. He was instrumental in establishing the development and regulatory strategies for the approvals of 74 new drug applications, nine biologics license applications, 28 abbreviated new drug applications, and many prescription-to-over-the-counter switch strategies. He was senior vice president for global compliance at Schering-Plough Corporation until his retirement in January 2004. He served as a member and chairman of the regulatory affairs coordinating committee of the Pharmaceutical Research and Manufacturers Association.

He was one of the original members of the steering committee of the International Council for Harmonisation of Technical Requirements for Pharmaceuticals for Human Use (ICH) and served as one of the two US pharmaceutical industry representatives from 1990 to 2003. While serving on the ICH steering committee, he introduced the concept of the Common Technical Document and served as co-chair of the Common Technical Document Implementation Coordination Group and the Global Cooperation Group.

Dr Giaquinto is a member of the American Association of Pharmaceutical Sciences, the American Society of Clinical Pharmacology and Therapeutics, and the New York Academy of Sciences. He has lectured in food and drug law for Temple University’s graduate program in Quality Assurance/Regulatory Affairs. Dr Giaquinto is a member of the board of directors for Biothera Holding Corporation and Biothera Pharmaceuticals Inc, both of Minneapolis. He has also held board positions with SemBioSys Genetics and Protox Therapeutics, both of Canada, and CannaPharmaRx of New Jersey.

**Alberto Grignolo, PhD,** Corporate Vice president at Parexel

Dr Alberto Grignolo is currently a Corporate Vice President at Parexel International, where he has worked for the past 27 years, and most recently established the firm’s Japan Consulting Services during a two-year assignment in Tokyo. He participates actively in Parexel’s Diversity and Inclusion initiatives and in corporate efforts to promote and maintain a High-Performance Culture and excellent customer service.

He was previously President of FIDIA Pharmaceutical Corporation and held positions in regulatory affairs at Smith Kline & French Laboratories. He has served as an adviser to the Institute of Medicine of the National Academy of Sciences on human subject protection in clinical trials, and he is a former member of the first Executive Committee of the Clinical Trials Transformation Initiative (CTTI). Dr Grignolo has served as a leader of several industry organizations, including as Chair of the DIA Board of Directors, and Chairman of the Regulatory Affairs Professionals Society.

A member and volunteer with DIA for over 30 years, Dr Grignolo serves on the faculty for DIA’s *Regulatory Affairs: The IND, NDA, and Postmarketing*training course and has taught this course numerous times in Japan, China, Korea, Europe, and the United States for more than a decade. Dr Grignolo has been a frequent Speaker, Program Chair, Session Chair or Instructor at international conferences, seminars, workshops, and courses on Drug Development and Regulatory Affairs. He was Program Chair of the *DIA 2007 Annual Meeting* and is currently the Editor-in-Chief of *Global Forum*, DIA’s digital magazine. He received DIA’s *Global Inspire Award* (Global Connector) in 2015 and was named a DIA Fellow in 2017.

**John CW Lim, MD**, Founding Executive Director of the Centre of Regulatory Excellence at the Duke–National University of Singapore (NUS) Medical School and Policy Core Lead of the SingHealth Duke-NUS Global Health Institute

A medical doctor with graduate degrees in Public Health from NUS and Health Policy and Management from Harvard University, Dr Lim is Professor of Practice at Duke-NUS and the NUS Saw Swee Hock School of Public Health, Senior Advisor at Singapore’s Ministry of Health (MOH), and Chairman of the Singapore Clinical Research Institute and National Health Innovation Centre.

Formerly Chief Executive Officer of Singapore’s Health Sciences Authority and Deputy Director of Medical Services (Industry & Research Matters) in MOH, Professor Lim has also held other senior positions in the health and education ministries. In his current roles, he draws on his international experience and networks to enhance health regulatory and systems capacity and scientific excellence for national authorities, industry and researchers in the Asia-Pacific and South-East Asia.

He is a member of the Executive Board of the Asia-Pacific Economic Cooperation Life Sciences Innovation Forum, Advisory Group of the US Pharmacopoeia’s Quality Institute, Scientific Advisory Council of the Centre for Innovation in Regulatory Science, Association of Southeast Asian Nations Diagnostics Development Initiative Strategic Planning Panel, Board of the Singapore Food Agency, and Board of St Andrew’s Mission Hospital in Singapore.

In 2018, Professor Lim received the Drug Information Association’s Global Connector Inspire Award for leadership in promoting global collaboration to advance healthcare products to patients, and the Regulatory Affairs Professional Society’s highest Founder’s Award recognising substantial sustained impact in shaping regulatory practice and policy over the course of his career.

**Larry Liberti, PhD**, Head of Regulatory Collaborations at the Centre for Innovation in Regulatory Science (CIRS)

Dr Liberti has worked in the fields of pharmaceutical regulatory affairs, communications, and clinical R&D for the past four decades. He began his career at Wyeth Laboratories working in product development, then as a regulatory writer in clinical R&D and manager of safety surveillance in medical affairs. He served as the editorial director for the North American operations of ADIS International, after which he founded PIA Ltd, a company specializing in regulatory writing and consulting; he co-founded Astrolabe Analytica under which he helped develop, patent and commercialize the Astrolabe Message Mapping System.^TM^

From 2009 to 2019 he has served as the Executive Director of the Centre for Innovation in Regulatory Science (CIRS), Ltd, forming part of Clarivate Analytics. In 2019 he transitioned to his current position. He has been actively involved in promulgating best practices in the regulatory aspects of medicines development, especially in the emerging markets.

Dr Liberti received his doctorate in International Regulatory Policy through the WHO Collaborating Centre for Pharmaceutical Policy and Regulation based in the Utrecht Institute for Pharmaceutical Sciences, Utrecht University, the Netherlands, where his research centered on expedited regulatory pathways with applicability in the emerging markets.

He is a Fellow of the American Medical Writers Association and is a recipient of their Golden Apple award for excellence in teaching. Dr Liberti is a co-founder of the Erudee Foundation, a non-profit dedicated to supporting local post-secondary educational opportunities for students in the Caribbean and East Africa.

**Tomas Salmonson, PhD**, partner at Consilium Salmonson & Hemmings

Dr Salmonson left the Medical Products Agency in February 2019 after more than 30 years at the Swedish agency and the European regulatory network.

Dr Salmonson chaired the Committee for Medicinal Products for Human Use (CHMP) at the European Medicines Agency (EMA) between 2012 and 2018. Before that, he was a member of CPMP/CHMP representing Sweden between 1999 and 2012. During the last ten years at CHMP, he also represented the European Union (EU) at the ICH Steering /Management Committee and the ICH Assembly.

**Fernand Sauer, MPharm,** Founder and first Executive Director of the European Medicines Agency

Fernand Sauer is a member of the French Academy of Pharmacy. He joined the European Commission in 1979 and was involved in the completion of the European internal market for pharmaceuticals, the EU accession to the European Pharmacopoeia Convention, and the development of industrial policy in the sector. He played a key role in launching the trilateral harmonization of regulatory requirements (ICH) between Europe, the United States, and Japan.

He created the EMA and became its first Executive Director in London from 1994 to 2000. He put in place a fast, effective, and transparent scientific evaluation system for innovative human and veterinary medicines, in particular “orphan drugs” for rare diseases.

As director for public health of the European Commission (2000 to 2005), he was responsible for the first European public health program; for the quality and safety of blood, tissues, and cells; for tobacco control; and for responses to threats from communicable diseases and bioterrorism. He participated in the creation of the European Centre for Disease Prevention and Control (ECDC) in Stockholm in 2005.

As a member of the College of the High Council for Public Health (2007 to 2011), he contributed to the evaluation of the French public health legislation, with a special interest for rare diseases, antibiotic resistance, and emerging diseases.

He provided independent expertise for European research activities: impact assessment of the Innovative Medicines Initiative (IMI, 2006), two evaluation reports of the European and Developing Countries Clinical Trials Partnership Program on AIDS, TB and Malaria (EDCTP 2007 and 2009), and on the Future of European Public Health Research (2013).

**Henrietta Ukwu, MD,** **FACP, FRAPS,** Senior Vice President and Global Head of Global Quality & Global Regulatory Affairs at Otsuka Pharmaceutical Development & Commercialization, Inc

Dr Ukwu is a physician, infectious diseases expert, professor, pharmaceutical industry executive, thought leader, and author of the landmark textbook, *Global Regulatory Systems – A Strategic Primer for Biopharmaceutical Product Development and Registration*. Among many awards, Dr Ukwu is included on the 2019 Top Blacks in Health Care in the USA and the 2011 PharmaVoice 100 list of most inspiring industry leaders, and she received the November 2012 TOPRA award for Most Inspiring Leaders.

Dr Ukwu is currently Senior Vice President and Global Head, Global Quality and Global Regulatory Affairs, at Otsuka Pharmaceutical Company, which she joined in 2013. In this dual executive role, Dr Ukwu is responsible for driving regulatory strategic and operational excellence, as well as quality compliance excellence and inspection readiness for Otsuka product development, registration, and maintenance.

Dr Ukwu has over two decades of executive pharma Industry leadership including at Merck, Pfizer, and Pharmaceutical Product Development (PPD), where she held Vice President/Senior Vice President positions leading Global Regulatory teams.

Dr Ukwu has extensive experience in global regulatory and quality, with outstanding performance and results. She has led numerous successful global regulatory product development activities across all regions and is directly responsible for over 25 original and key product approvals across multiple therapy areas and platforms. Dr Ukwu has led major transformation initiatives to drive regulatory excellence and redefined the role of Regulatory Affairs and Quality teams as an interactive, intellectual, strategic business partner and company resource and enabler to achieve significant business success and public health goals. Furthermore, Dr Ukwu has established remarkable expertise in driving successful globalization and transformation of regulatory and quality functions to high performing and outcomes- based organizations.

Dr Ukwu serves on company executive and senior leadership and governance committees.

Dr Ukwu has made significant contributions to medicine, science, and pharmaceutical industry, including developing and enriching the regulatory and quality profession, as well as producing many protégés in senior leadership positions in industry. She has received numerous awards and recognition for these contributions, including Fellow of the Regulatory Affairs Professional Society (FRAPS) and Fellow of the American College of Physicians (FACP), as well as many company prestigious awards. Dr Ukwu is a pioneer professor of Temple University’s Quality Assurance/Regulatory Affairs graduate program.
